# Big Five Personality Factors Differentially Related to Positive and Negative Affect Intensity of Autobiographical Memories

**DOI:** 10.1111/sjop.70039

**Published:** 2025-10-30

**Authors:** Sophie Hoehne

**Affiliations:** ^1^ Department of Developmental Psychology, Institute of Psychology and Education Ulm University Ulm Germany

**Keywords:** affect intensity, autobiographical memory, big five personality traits, exploratory factor analysis, multilevel modeling

## Abstract

Although the relationship between personality and aspects of emotional experience have been investigated from a variety of different perspectives, little research has been conducted on personality traits and the affect intensity of autobiographical memories (AMs). To fill this gap, the present study examined the association of the Big Five personality traits with the positive and negative affect intensity of positive and negative AMs using multilevel multiple regression. Participants (*N* = 1275; 18–53 years) completed the short form of the Big Five Inventory (BFI‐K) and reported AMs of three positive and three negative events. Next, participants rated the negative and positive affect intensity of each event separately on a 7‐point Likert scale. Neuroticism was associated with less positive and more negative affect intensity of both positive and negative AMs. Extraversion showed the opposite pattern with respect to positive AMs, and agreeableness with respect to negative AMs and the negative affect intensity of positive AMs. Openness was associated with a higher positive affect intensity of positive AMs and conscientiousness with a higher positive affect intensity of negative AMs. Results are discussed in relation to previous empirical evidence and theoretical considerations of the associations. The present study extends previous research by demonstrating that all Big Five traits relate to the affect intensity of individuals' AMs.

**Summary**

- The present study demonstrates that all Big Five traits are related to the affect intensity of individuals' autobiographical memories
- Neuroticism was associated with a lower positive affect intensity and higher negative affect intensity of individuals' positive and negative autobiographical memories
- Extraversion was associated with a higher positive and a lower negative affect intensity of individuals’ positive autobiographical memories
- Openness was associated with a higher positive affect intensity of individuals' positive autobiographical memories
- Agreeableness was associated with a higher positive affect intensity of individuals' negative autobiographical memories and a lower negative affect intensity of individuals' positive and negative autobiographical memories
- Conscientiousness was associated with a higher positive affect intensity of individuals' negative autobiographical memories

Although various studies have found associations between personality traits and emotional experience (e.g., Anglim et al. 2020; Barańczuk 2019; Olaru et al. 2023), few studies to date have examined the relationship between personality traits and the affect intensity of autobiographical memories (AMs). However, from a theoretical perspective, there may be several potential pathways at different stages of the memory, namely encoding, (re)consolidation and recall, through which personality traits could be related to the affect intensity of AMs (e.g., Adler and Pansky 2020). It is therefore surprising that only neuroticism, and in some respects extraversion, but not openness, agreeableness and conscientiousness, have been systematically studied in relation to the affect intensity of AMs. The goal of the present study was to fill this gap in personality research by systematically examining the relationship between all of the Big Five traits and the positive and negative affect intensity of positively and negatively valenced AMs in a large sample of younger and middle‐aged adults.

## 1 Personality Traits, Emotions and Autobiographical Memory

Personality can be described as a specific pattern of feelings, actions and thoughts that is relatively stable across time and situations (McCrae and Costa 2003). Various theories of personality have been proposed but one of the most influential and most commonly used taxonomies of personality traits is the Five Factor Model of Personality, also known as the “Big Five” (Costa and McCrae 1992). The Big Five model proposes that people differ in terms of five broad domains of personality, namely, neuroticism (or its converse, emotional stability), extraversion, agreeableness, openness to experience, and conscientiousness (Costa and McCrae 1992).

Emotions typically arise in response to specific (external or internal) stimuli, such as conversations or simple everyday situations (external), but also when recalling autobiographical memories (internal). As they arise, emotions are associated with changes in subjective experience, behavior, and peripheral physiology relevant to the stimuli (Gross 2015).

To capture the relationship between personality and emotions, Revelle and Scherer (2009) used the metaphor of the relationship between climate and weather. Specifically, whereas personality is characterized by relatively stable tendencies to feel, act and think, emotion integrates feelings, actions and thoughts, but in a specific moment and place (Revelle and Scherer 2009). In line with this, a large meta‐analysis (Anglim et al. 2020) found that higher levels of neuroticism were associated with the experience of more negative emotions and less positive emotions in the past few weeks, whereas extraversion and conscientiousness were associated with more positive emotions. Empirically, the relationship between the Big Five and aspects of emotional experience has also been investigated from a variety of other perspectives, such as in the context of emotion regulation Barańczuk (2019) or life satisfaction Olaru et al. (2023). As argued above, emotions can be triggered also by the recall of autobiographical memories (AMs). However, little research has examined how individual differences in personality relate to the emotional experience based on AMs, that is, the intensity of positive and negative affect associated with the memories of specific life events.

Autobiographical memory can be broadly described as memories of life events that occurred at a specific time and place, and form an individual's life story (e.g., Fivush 2011; Tulving 2001). The relevance of autobiographical memory for the study of personality traits lies not only in its ability to trigger emotions, but also in its close and reciprocal connection to the self. Specifically, according to the self‐memory system model (Conway 2005), the self is predominantly defined by its autobiographical knowledge base, but what is remembered in the long term is, in turn, defined by the self.

## 2 Theoretical Considerations: Different Pathways to Emotional Memory

The relationship between personality traits and the affect intensity of AMs may arise in different ways. Specifically, from a theoretical perspective, personality traits may be related to the positive and negative affect intensity of AMs at different stages of the memory, that is encoding, (re)consolidation and recall (for a similar argumentation see, Adler and Pansky 2020).

### 2.1 Experiencing and Encoding Emotional Events

Olaru et al. (2023) argued that personality relates to life satisfaction through two different pathways, a direct and an indirect pathway. The direct path represents the link between individuals' personality traits and their general view of life and the world, whereas the indirect path represents the link between individuals' personality traits and their general disposition to experience positive and negative events. Through these two pathways, personality may also be related to autobiographical memory: The events that individuals experience in their daily lives form the basis of what might become their autobiographical memory (indirect path) and the ‘lenses’ through which individuals construe these events determine how these events are encoded (direct path).

With respect to the Big Five, Klaiber et al. (2022) found that participants scoring high on openness and/or extraversion were more likely to report having experienced positive events (indirect path). In addition, Jonason and Sherman (2020) found that individuals who differed in the Big Five perceived situations presented through pictures (bar, classroom, office) differently (direct path). Participants high in neuroticism perceived more threat and conflict in the situations, whereas participants high in agreeableness and extraversion perceived more social aspects, and participants high in conscientiousness and openness perceived more safety. The association between personality traits and reactivity to emotional stimuli has also been demonstrated in neurological research (Adelstein et al. 2011; DeYoung et al. 2010). For instance, have higher levels of extraversion been associated with greater amygdala activation during the presentation of positive pictures compared to negative ones (Canli 2004). Note that the influence of the two pathways on the affect intensity of AMs is strongly interrelated, as whether a person experiences positive or negative events with a high or low intensity depends not only on the *objective* content of the events, but also on the *subjective* experience of the individual.

### 2.2 Regulating Emotional Events and (Re)consolidating Emotional Memories

Barańczuk (2019) found in a meta‐analysis of 132 studies that higher levels of extraversion, conscientiousness, agreeableness, and openness, but lower levels of neuroticism, were positively associated with the use of more adaptive emotion regulation strategies and negatively associated with the use of more maladaptive emotion regulation strategies. Moreover, Wisco and Nolen‐Hoeksema (2010) found that dispositions to specific emotion regulation strategies were related to the affect intensity of individuals' AMs. Specifically, individual levels of reappraisal (adaptive emotion regulation strategy) were associated with a higher positive intensity of participants' AMs, and individual levels of suppression (maladaptive emotion regulation strategy) were associated with a faster retrieval of negative AMs.

Emotion regulation dispositions may be relevant to the affect intensity of individuals' AMs in two ways. First, how an event is emotionally regulated *during* the event may influence how it is encoded in terms of valence and affect intensity (Wisco and Nolen‐Hoeksema 2010), and second, how the memory of an event is regulated *post hoc* can influence its (re)consolidation and future accessibility (Hoehne and Zimprich 2024, 2025a; Nørby 2019). To illustrate, Muir et al. (2022) found that participants *high* in neuroticism thought about their negative memories more frequently and reported that the affect intensity of these negative memories remained comparatively stable from the moment of event occurrence to the moment of recall, whereas participants *low* in neuroticism recalled unpleasant events less frequently and reported that the affect intensity they associated with these negative memories had faded more strongly from the moment of event occurrence to the moment of recall (see also, Hoehne and Zimprich 2025b).

### 2.3 Recalling Emotional Events

Finally, personality traits may be related to the accessibility of AMs through differences in dispositional affect. A number of studies have found that an individual's dispositional affect is associated with their personality traits, with particular emphasis on neuroticism (dispositional negative affect) and extraversion (dispositional positive affect) (e.g., Anglim et al. 2020; Haas and Canli 2008; Shiota et al. 2006). Regarding the other Big Five dimensions, Shiota et al. (2006) found that openness correlated with feelings of joy, love, amusement, awe, and compassion, whereas agreeableness correlated with a disposition to experience empathic positive emotions, and conscientiousness correlated with joy, contentment and pride. Haas and Canli (2008) found that a tendency to regularly experience certain affective states is related to facilitated retrieval of emotionally congruent memories. This link has also been demonstrated in neuroimaging studies (for a review, see Faul and LaBar 2022).

Taken together, personality traits may be linked to the affect intensity of AMs through different pathways and mechanisms that occur at different stages of the memory, namely encoding, (re)consolidation, and recall. The following predictions emerge: High levels of neuroticism may be associated with increased negative affect intensity and decreased positive affect intensity of individuals' AMs, whereas high levels of extraversion may be associated with increased positive affect intensity and decreased negative affect intensity of individuals' AMs. In addition, high levels of conscientiousness, agreeableness, and openness may also be associated with a tendency of increased positive affect intensity and decreased negative affect intensity of individuals' AMs, mostly through their association with adaptive emotion regulation strategies (Barańczuk 2019), and dispositional positive affect (Shiota et al. 2006), but also through their disposition to experience and encode positive and negative events (Jonason and Sherman 2020; Klaiber et al. 2022).

## 3 Previous Empirical Evidence on the Big Five and Emotional Autobiographical Memory

To date, few studies have empirically investigated the relationship between personality traits and the affect intensity of AMs. In a sample of 62 younger adults, Denkova et al. (2012) examined how individuals high in neuroticism and extraversion differed in the likelihood of having experienced highly intense positive and negative events. Denkova et al. (2012) found that, across participants, higher levels of extraversion were associated with a greater number of highly intense positive AMs, whereas neuroticism was associated with a greater number of highly intense negative AMs in men. Denkova et al. (2012) excluded all non‐high‐intensity AMs from the analyses, so it remains unclear how neuroticism and extraversion relate to other than high‐intensity AMs.

More recently, Nusser et al. (2024) investigated the predictive value of extraversion, neuroticism and openness on the affect intensity (measured on one scale from negative to positive) of word‐cued and important AMs in a sample of 104 older adults. They found that extraversion was associated with more positive/less negative affect intensity of word‐cued but not important AMs, whereas neuroticism was associated with more negative/less positive affect intensity of word‐cued and important AMs. Openness had no effect, and the predictive value of agreeableness and conscientiousness was not investigated.

Blagov et al. (2022), who examined the association between the Big Five traits and other personality traits with features of self‐defining AMs in a sample of 133 undergraduates, examined all of the Big Five traits and separately analyzed positive and negative intensity of each AM. They found that extraversion was associated with a more intense positive affect for participants' self‐defining AMs, whereas neuroticism and conscientiousness were associated with less intense positive affect, and neuroticism with more intense negative affect for participants' self‐defining AMs. Blagov et al. (2022) did not examine whether individuals differed in other than self‐defining AMs.

Note that the above studies differ in that they measured the affect intensity of AMs either on a single scale from negative to positive, or on two scales, one for positive and one for negative affect intensity. There is an ongoing debate about the relative independence of positive and negative affect. More recent research has found that the degree of independence of positive and negative affect varies within individuals and between situations. For instance, Dejonckheere et al. (2021) found that higher personal importance of an event was associated with greater bipolarity of positive and negative affect and, conversely, that a longer time since an event was associated with greater independence (see also Diener and Emmons 1985). With regard to AMs, it appears that positive and negative affect intensity represent distinctly different dimensions of human emotional experience and can both be associated with the same event at the same time (e.g., Hoehne 2023).

## 4 The Present Study

In summary, there are, from a theoretical point of view, several pathways through which personality traits might be linked to the affect intensity of AMs, such as differences in experiences, encoding, emotion regulation, or dispositional affect. With respect to neuroticism and extraversion, previous empirical studies broadly support the predictions derived from this theoretical perspective. However, no study to date has examined this relationship in the context of all of the Big Five traits, using a more unrestricted memory recall procedure, and a large sample size. The aim of the present study was to fill this gap by examining the relationship between all Big Five traits and both the positive *and* negative affect intensity of positive *and* negative AMs retrieved in a valence‐cued but otherwise free recall procedure in a large sample. Based on the presented theoretical and empirical evidence, it was predicted that neuroticism would be associated with increased negative affect intensity and decreased positive affect intensity in individuals' AMs, whereas extraversion, conscientiousness, agreeableness, and openness would be associated with increased positive affect intensity and decreased negative affect intensity.

## 5 Method

### 5.1 Sample

From the existent data set comprising 2803 persons who started (but did not necessarily complete) the online study, those 604 persons who did not provide basic demographic data (age, gender) were excluded. Next, those 338 individuals who did not answer all five catch items correctly and/or reported to not have answered carefully and honestly were excluded. From the remaining 1861 persons, only those individuals who answered all BFI‐K items were included, which reduced the sample by 167 persons (146 participants had missing data on all 21 BFI‐K items. The main reason for missingness probably was that the random ordering of tests put the BFI‐K into the last position of the online study for 133 of these 146 participants). Next, only those who provided all six AMs plus the according affect intensity ratings were included, reducing the sample by 419 participants. Of these 419 persons, most had missing values for the third positive (*N* = 318) and/or the third negative AM (*N* = 358).^1^

The final sample comprised *N* = 1275 adults between the ages of 18 to 53 (M = 33.65, SD = 10.76).^2^ Women were overrepresented (64.86% women, 0.94% gender diverse, 0.47% who did not want to disclose their gender), as well as participants who indicated that German was their first language (94.20%), and who had a university entrance diploma (82.75%).^3^ Participants were recruited through word‐of‐mouth, email, and promotional flyers. Inclusion criteria were good knowledge of German language and an age of at least 18 years. After taking part in the study, participants could choose to participate in a lottery to win a voucher (worth 50, 30 or 20 Euros).

### 5.2 Procedure

The study's design and its analysis were not pre‐registered. The data for the present study were collected online between November 2023 and February 2024 as part of a large non‐experimental project. The project was conducted in accordance with the Declaration of Helsinki. Participants completed the study via SoSci Survey (Leiner 2024) (www.soscisurvey.de). After being presented with the study information and signing the informed consent form, participants provided subjective health (physical and mental) and demographic information (age, gender, education, marital status, first language, population of their place of residence, whether they lived alone). They were then administered several individual difference questionnaires and an autobiographical memory task in a randomized order (for more details see ‘Measures’). Within each questionnaire, all items were also presented in a completely randomized order. One of the individual difference questionnaires was the short form of the Big Five Inventory (BFI‐K) (Rammstedt and John 2005), which measures the Big Five personality traits.^4^ Finally, participants were asked whether they had answered all the questions carefully and honestly.

### 5.3 Measures

#### 5.3.1 The Big Five Personality Traits

The BFI‐K (Rammstedt and John 2005) consists of 21 items and is an abbreviated version of the Big Five Inventory (John et al. 1991). In the present study, the German version of the scale (Rammstedt and John 2005) was used. Previous research has shown that the BFI‐K has good psychometric properties and is a reliable and valid instrument for measuring the Big Five personality traits in an economic time frame (Kovaleva et al. 2014; Rammstedt and John 2005). Participants were asked to respond to each item on a 7‐point Likert‐type scale ranging from strongly disagree (1) to strongly agree (7). The Neuroticism, Extraversion, Agreeableness, and Conscientiousness subscales each consist of four items, and the Openness subscale comprises five items. An example item for extraversion is: “I see myself as someone who is outgoing, sociable.”, whereas a negatively worded example item for agreeableness is: “I see myself as someone who is sometimes rude to others.” For extraversion, openness, agreeableness, and conscientiousness, all negatively worded items were reverse scored, whereas for neuroticism all positively worded items were reverse scored.

Regarding the factorial structure of the BFI‐K, some researchers have argued that instead of a congeneric structure (where every item loads on its designated factor only) secondary loadings should be allowed (Kovaleva et al. 2014; Marsh et al. 2010; McCrae et al. 1996). As a result, Confirmatory Factor Analysis (CFA)—usually the tool of choice when examining the factor structure of a scale—is considered less appropriate for assessing the factor structure of personality inventories (e.g., Kovaleva et al. 2014). Therefore, similar to Rammstedt and John (2005), an Exploratory Factor Analysis (EFA) was conducted, which allows items to have meaningful (secondary) loadings on more than one factor, to obtain the factor structure of the BFI‐K in the present data.

Correlations between all 21 BFI‐K items are presented in Table S1. Before submitting the correlation matrix of the 21 BFI‐K items for EFA, their factorability was tested. Bartlett's test of sphericity (Bartlett 1950) indicated that the correlation matrix was adequate for factor analysis $\left(x^{2}\left(210\right)=9328,p<.001\right)$, and the Kaiser‐Meyer‐Olkin (KMO) statistic (Kaiser 1974) was 0.79, which is substantially above the criterion for conducting factor analysis (Hoelzle and Meyer 2013), thus validating the sampling adequacy for the analysis. Common factor analysis was chosen due to the current goal of identifying a latent structure (Watkins 2018). The extraction method was principal factors, and the initial communalities were estimated by squared multiple correlations. Theory (e.g., Rammstedt and John 2005), the Scree test (Cattell 1966), and Kaiser's criterion of eigenvalues greater than 1 (e.g., Stevens 1992) all suggested that a five‐factor solution represented the best fit for the data. Parallel to the original validation study of the BFI‐K (Rammstedt and John 2005), an orthogonal rotation (varimax) was chosen, which implies no correlation between the extracted factors. A cut‐off value for salient factor loadings was set a priori at 0.32, meaning that at least 10% (≈0.32^2^) of the true variance in an item must be explained by a factor in order to be considered salient. Factors were considered adequate if they had at least three salient pattern coefficients and an internal consistency (Cronbach's alpha) of at least 0.60.^5^

The results indicated a simple structure (Thurstone 1947) of the data. Specifically, the five factors meaningfully represented the Big Five domains, with each item saliently loading on its designated factor, with no salient cross‐loadings. The rotated loading patterns are presented in Table 1. After rotation, the neuroticism factor accounted for 11% of the total variance and 23% of the common variance, whereas the extraversion factor accounted for 11% of the total variance and 24% of the common variance, the openness factor for 10% of the total variance and 20% of the common variance, the agreeableness factor for 8% of the total variance and 16% of the common variance, and the conscientiousness factor for 8% of the total variance and 17% of the common variance. Coefficients' alpha were 0.83 for the neuroticism factor, 0.84 for the extraversion factor, 0.75 for the openness factor, 0.69 for the agreeableness factor, and 0.71 for the conscientiousness factor.

**TABLE 1:** Varimax‐rotated five‐factor structure of the BFI‐K items (*N* = 1275).

BFI‐K item	Neuroticism	Extraversion	Openness	Agreeableness	Conscientiousness
*Extraversion*					
Item 1	—	**0.85**	—	—	—
Item 6	—	**0.50**	0.29	—	0.23
Item 11	—	**0.82**		—	
Item 16	—	**0.72**		—	
*Neuroticism*					
Item 4	**0.73**	—	—	—	—
Item 9	**0.67**	—	—	—	—
Item 14	**0.73**	—	—	—	—
Item 19	**0.71**	−0.26	—	—	—
*Openness*					
Item 5	—	—	**0.54**	—	—
Item 10	0.22	—	**0.45**	—	—
Item 15	—	—	**0.63**	—	—
Item 20	—	—	**0.69**	—	—
Item 21	—	—	**0.72**	—	—
*Agreeableness*					
Item 2	—	—	—	**0.53**	—
Item 7	—	—	—	**0.33**	—
Item 12	—	—	—	**0.70**	—
Item 17	—	—	—	**0.76**	—
*Conscientiousness*					
Item 3	—	—	—	—	**0.63**
Item 8	—	—	—	—	**0.55**
Item 13	—	—	—	—	**0.65**
Item 18	—	—	—	—	**0.56**

*Note:* Absolute values smaller than 0.2 (less than approximately 5% variance explained) are not shown. Values above 0.32 (= approximately 10% variance explained) were considered salient and are printed in bold.

The loading pattern is quite similar in size and structure to that found by Rammstedt and John (2005) in the original validation study of the BFI‐K. Only the amount of total variance explained in the present data was somewhat lower than that found by Rammstedt and John (2005), while internal consistencies of the five factors were slightly larger.

### 5.4 Autobiographical Memory Task

Prior to the autobiographical memory task, participants were instructed that the memories to be recalled did not necessarily have to be extraordinary, but that each one should relate to a specific and distinct event from their past and that the event should be older than 1 year (exact wording of the instructions in the online Supporting Information).^6^ For the actual autobiographical memory task, participants were asked to freely recall three AMs of events that they perceived as overall more positive at event occurrence, hereafter referred to as *positive AMs*, and three AMs of events that they perceived as overall more negative at event occurrence, hereafter referred to as *negative AMs* (“Please describe in a few words an event from your past that you experienced as overall more [positive/negative] at the time it happened.”). Participants provided brief descriptions of each event so that they would be able to recall it when asked questions about it (see below). Whether participants were asked to describe the positive or negative events first was randomized, so that participants described either the three positive events first—one at a time –, or the three negative events. Randomization was employed to avoid any carry‐over effects. After each event description, participants were asked to answer a series of questions relating to the event. Questions included the *affect intensity* of each event from the perspective at recall, that is, from the perspective of now.^7^ Unlike ratings from the perspective of an event at the time it happened, ratings from a current perspective are also based on previous emotional regulation and more accurately represent how the individual actually remembers and evaluates the event. The ratings were made separately for positive and negative affect intensity for each event (“How [positive/negative] would you rate the event emotionally today?”). Responses were made on a Likert‐type scale ranging from *not at all* (1) to *very much* (7).

## 6 Statistical Approach

The goal of the present study was to examine the predictive value of each of the Big Five traits on the positive and negative affect intensity of positive and negative AMs. Because AMs are nested within participants (each participant reported up to 6 AMs), resulting in a two‐level structure of the data, the multiple regression model was extended to include random intercept effects (Bryk and Raudenbusch 1992; Hedeker and Gibbons 2006).^8^ The random intercepts allow for the fact that individuals differ in terms of the average positive and negative affect intensity of the positive and negative AMs they report. Before running the multilevel regression model, however, it was necessary to determine the factor structure of the BKI‐K and to extract the resulting factors. For this purpose, an Exploratory Factor Analysis (EFA) (Fabrigar et al. 1999; Watkins 2018) was conducted (see above).^9^ The extracted factors for each Big Five domain from the EFA were then centered around the sample mean and used as predictors of the positive and negative affect intensity of participants' positive and negative AMs in the multilevel multiple regression models. Separate analyses were conducted for each affect intensity outcome (positive intensity of positive AMs, negative intensity of positive AMs, positive intensity of negative AMs, and negative intensity of negative AMs). Moreover, in order to adjust for their possible effects, age, gender, and education (whether a participant had a university entrance diploma) were centered around the sample mean and included as control variables in the analyses.^10^ All analyses were conducted using SAS Proc Calis (SAS Institute Inc. 2014).

## 7 Results

### 7.1 Descriptive Statistics

Means and standard deviations of all BFI‐K and AM affect intensity items are shown in Table 2. Autobiographical memories reported in response to the instruction to recall positive events, were, on average, rated as relatively high in positive affect intensity and low in negative affect intensity, while those reported in response to the instruction to recall negative events were, on average, rated as relatively high in negative but low in positive affect intensity, indicating that the manipulation in the present study was successful. Scale means of all Big Five domains are highly similar to those reported by Rammstedt and John (2005) as well as Kovaleva et al. (2014). Specifically, the lowest average mean scores were found for the neuroticism items, suggesting that the average neuroticism score in the present sample was the lowest of all the Big Five domains. In contrast, the highest mean scores were found for openness, indicating that this domain was relatively strong in the present sample. All the other Big Five domain means were also moderately to relatively high in the present sample.

**TABLE 2:** Means and standard deviations for all items (*N* = 1275).

Item	Mean	Std	Item	Mean	Std
*Positive autobiographical memories (PAM)*			*Negative autobiographical memories (NAM)*		
Positive affect PAM1	6.47	1.00	Positive affect NAM1	2.63	1.86
Negative affect PAM1	1.49	1.05	Negative affect NAM1	5.24	1.74
Positive affect PAM2	6.20	1.38	Positive affect NAM2	2.76	1.94
Negative affect PAM2	1.76	1.42	Negative affect NAM2	4.90	1.94
Positive affect PAM3	6.06	1.53	Positive affect NAM3	2.89	1.93
Negative affect PAM3	1.94	1.59	Negative affect NAM3	4.81	1.91
*BFI‐K*: *neuroticism*			*BFI‐K: extraversion*		
BFI‐K item 4	3.74	1.65	BFI‐K ITEM 1^a^	4.38	1.67
BFI‐K item 9^a^	3.90	1.58	BFI‐K item 6	5.15	1.39
BFI‐K ITEM 14	4.64	1.63	BFI‐K item 11^a^	4.66	1.72
BFI‐K item 19	4.03	1.65	BFI‐K item 16	4.76	1.54
*BFI‐K: openness*			*BFI‐K: agreeableness*		
BFI‐K item 5	5.72	1.20	BFI‐K item 2^a^	4.17	1.48
BFI‐K item 10	5.70	1.27	BFI‐K item 7	4.99	1.45
BFI‐K item 15	5.30	1.42	BFI‐K item 12^a^	3.83	1.67
BFI‐K item 20	5.27	1.52	BFI‐K item 17^a^	4.09	1.67
BFI‐K item 21^a^	4.68	1.74			
*BFI‐K: conscientiousness*					
BFI‐K item 3	5.53	1.13			
BFI‐K item 8^a^	4.28	1.66			
BFI‐K item 13	5.27	1.21			
BFI‐K item 18	5.21	1.20			

*Note:* All items were measured on a scale from 1 to 7. PAM1 refers to the first positive autobiographical memory, PAM2 to the second positive autobiographical memory, and so on.

^a^ The original item was reversed.

## 8 Multilevel Multiple Regression: Big Five Factors and the Affect Intensity of Autobiographical Memories

To examine the association of the extracted Big Five factors with the positive and negative affect intensity of participants' positive and negative AMs, multilevel multiple regression models were examined in the next step. First, baseline models (Model 0) were estimated for each of the four affect intensity variables, including only random intercepts. The corresponding parameter estimates are shown in Table 3. All random intercept variances were significant, indicating that participants reliably differed in their mean positive and negative affect intensity ratings of positive and negative AMs. The intraclass correlation for the positive affect intensity of positive AMs was 0.26, indicating that 26% of the variance in the positive affect intensity of positive AMs reflected differences between participants. Similarly, the intraclass correlation for the negative affect intensity of positive AMs was 0.28, for the positive affect intensity of negative AMs 0.21, and for the negative affect intensity of negative AMs 0.19.

**TABLE 3:** Multivariate predictor parameter estimates for positive and negative affect intensity of participants' positive and negative autobiographical memories (*N* = 1275).

	Positive AMs				Negative AMs			
	Positive intensity		Negative intensity		Positive intensity		Negative intensity	
	Model 0	Model 1	Model 0	Model 1	Model 0	Model 1	Model 0	Model 1
*Fixed effects*								
Intercept	6.24*	6.24*	1.73*	1.73*	2.76*	2.76*	4.98*	4.98*
*Control variables*								
Age^a^		0.00		0.00		−0.02*		0.02*
Gender^a^		0.20*		−0.03		−0.05		0.16*
University entrance diploma^a^		0.03		−0.09		0.02		−0.16^†^
*Predictor variables*								
Neuroticism^a^		−0.21*		0.28*		−0.12*		0.25*
Extraversion^a^		0.19*		−0.17*		0.02		−0.04
Openness^a^		0.09*		−0.05		0.05		0.02
Agreeableness^a^		0.03		−0.07*		0.10*		−0.16*
Conscientiousness^a^		0.01		0.02		0.10*		−0.06
*Random effects*								
Intercept variance	0.47*	0.39*	0.53*	0.44*	0.79*	0.74*	0.68*	0.56*
*Model fit*								
‐2LL	12,807	12,733	13,087	12,995	15,660	15,657	15,539	15,475
AIC	12,811	12,737	13,091	12,999	15,664	15,661	15,543	15,479

* *p* < 0.05 (two‐tailed).

^†^ *p* < 0.10 (two‐tailed).

^a^ Variable was centered around the sample mean.

In Model 1, control and predictor variables were added, resulting in an increase in model fit in all four models (see ‐2LL and AIC in Table 3). In the following, the results are presented separately for each Big Five factor (Table 3).

### 8.1 Neuroticism

**FIGURE 1:**
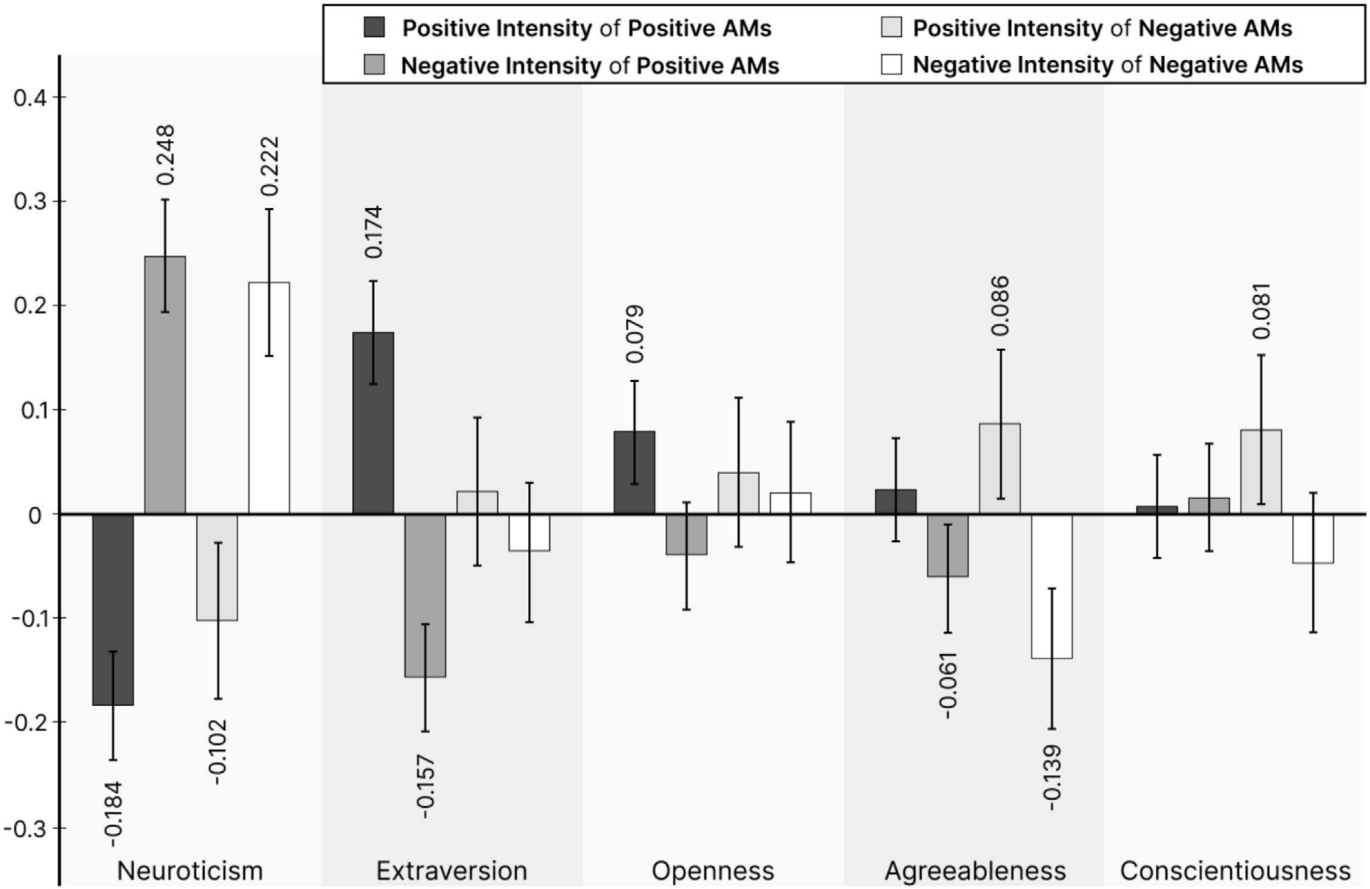
Standardized estimates from the multilevel multiple regression models with 95% confidence intervals.

The Neuroticism factor was significantly related to all four types of affect intensity. Specifically, participants with relatively higher Neuroticism reported both positive and negative AMs that were significantly lower in positive affect intensity and, simultaneously, higher in negative affect intensity compared to participants with relatively lower Neuroticism. Within the Neuroticism factor, the effects were stronger for negative affect intensity. The strongest effect occurred for the negative affect intensity of participants' positive AMs, and the smallest effect occurred for the positive affect intensity of participants' negative AMs. Compared to all other Big Five factors, the effects for Neuroticism were the largest in any single model. All standardized parameter estimates with 95% confidence intervals are also shown in Figure 1.

### 8.2 Extraversion

The Extraversion factor was only significantly related to the positive and negative affect intensity of participants' positive AMs. Specifically, higher Extraversion scores were associated with an increased positive affect intensity and a decreased negative affect intensity of participants' positive AMs. Both effects were of relatively equal magnitude. Within both models for positive AMs, the effects for Extraversion were the second largest after Neuroticism.

### 8.3 Openness

The Openness factor was only significantly related to the positive affect intensity of individuals' positive AMs. Participants with higher Openness scores reported positive AMs that were relatively higher in positive affect intensity than participants with lower Openness scores.

### 8.4 Agreeableness

The Agreeableness factor was significantly related to the negative affect intensity of participants' positive AMs and to both the positive and negative affect intensity of participants' negative AMs. Specifically, higher levels of Agreeableness were associated with lower negative affect intensity ratings of both participants' positive and negative AMs, and simultaneously with higher positive affect intensity ratings of their negative AMs. Within the Agreeableness factor, the effects were relatively larger for negative AMs, and the largest effect was found for the negative affect intensity of individuals' negative AMs.

### 8.5 Conscientiousness

The Conscientiousness factor was only significantly related to the positive affect intensity of participants' negative AMs. Participants with higher Conscientiousness scores reported negative AMs that were relatively higher in positive affect intensity compared to participants with lower Conscientiousness scores. The effect size was comparable to that of Agreeableness on the positive affect intensity of negative AMs.

## 9 Discussion

As autobiographical memory is closely connected to the self (Conway 2005), it is relevant to the study of personality traits. The main goal of the present study was to systematically examine the relationship between all of the Big Five factors and the positive and negative affect intensity of positive and negative AMs collected in a valence‐cued but otherwise free‐recall procedure in a large sample of younger and middle‐aged adults. Each of the Big Five factors showed its own unique pattern. The results are discussed below.

### 9.1 Affect Intensity of Autobiographical Memories

With respect to neuroticism, the present study provided further evidence for what has been found in previous studies, namely, that neuroticism is associated with less positive intensity and/or more negative intensity in AMs. Specifically, in the present study, higher levels of neuroticism were associated with a lower positive affect intensity and higher negative affect intensity of participant's positive and negative AMs, with relatively strong effects in all four models. Nusser et al. (2024), who measured affect intensity on a single scale, found that higher levels of neuroticism were associated with less positive affect intensity of participants' word‐cued and important AMs. In addition, Blagov et al. (2022), who examined the positive and negative affect intensity of individuals' AMs separately, as this study did, found that higher levels of neuroticism were associated with both less positive and more negative affect intensity of individuals' self‐defining AMs. Furthermore, Denkova et al. (2012), who examined the number of positive and negative AMs, found that higher levels of neuroticism were associated with a higher number of negative, high‐intensity AMs in men. Given that the present study replicated these patterns, but in a large sample and using a different methodology in terms of measures and the statistical approach, I consider the effect of neuroticism on the affect intensity of individuals' AMs to be a reliable and robust phenomenon.

Similarly, but not as consistently as for neuroticism, previous studies have found that extraversion is associated with more positive and/or less negative affect intensity in individuals' AMs. For instance, Nusser et al. (2024) found that higher levels of extraversion were associated with more positive affect intensity of participants' word‐cued but not important AMs, whereas Blagov et al. (2022) found that extraversion was associated with more positive affect intensity, but showed no effect on the negative affect intensity of individuals' self‐defining AMs. In contrast, in the present study, higher levels of extraversion were associated with a higher positive and a lower negative affect intensity, but only of individuals' positive AMs. Blagov et al. (2022) did not differentiate between positive and negative self‐defining AMs, which may explain why they did not find effects of extraversion on negative affect intensity. In this regard, the present study extends previous research by suggesting that extraversion may be particularly relevant to the affect intensity of individuals' positive, but not negative AMs.

Previous empirical evidence on the relationship between openness, agreeableness and conscientiousness with the affect intensity of individuals' AMs is rather scarce. With regard to openness, none of the studies that have examined this association have found a significant effect of openness (Blagov et al. 2022; Nusser et al. 2024). In the present study, however, openness exhibited a positive significant effect on the positive affect intensity of individuals' positive AMs. Importantly, however, the effect of openness was rather small.

Similar to the previous empirical evidence on openness, to the best of my knowledge, no study to date has found a significant effect of agreeableness on the affect intensity of individuals' AMs (Blagov et al. 2022). In contrast, the present study found a significant positive effect of agreeableness on the positive affect intensity of participants' negative AMs and significant negative effects on the negative affect intensity of both the positive and negative AMs of participants. In particular, the effect of agreeableness on the negative affect intensity of individuals' negative AMs was surprisingly strong, given that no other study to date has found significant effects of agreeableness on the affect intensity of individuals' AMs.

To date, and to the best of my knowledge, Blagov et al. (2022) is the only study to find an effect of conscientiousness on the affect intensity of individuals' AMs. Specifically, Blagov et al. (2022) found that higher levels of conscientiousness were associated with a lower positive affect intensity of individuals' self‐defining AMs. In contrast, the present study found that higher levels of conscientiousness were associated with a higher positive affect intensity, but only for participants' negative AMs. Blagov et al. (2022) did not examine the Big Five domains as latent factors, but operationalized them by calculating the means of the respective items. Consequently, and in contrast to the present study, the Big Five domains in Blagov et al. (2022) were correlated. The negative effect of conscientiousness on the positive affect intensity of individuals' AMs found by Blagov et al. (2022) might therefore be explained by the occurrence of suppression.^11^ From the theoretical perspective outlined in the introduction to this article, the present results, that is the *adaptive* effects of conscientiousness on the affect intensity of individuals' AMs, may be more coherent.

In general, the present results are largely consistent with the theoretical perspective outlined in the introduction to this article. Specifically, it was suggested that personality may be related to the affect intensity of individuals' AMs through several pathways at different stages of the memory, namely, individuals' general disposition to experience and encode specific events, how they regulate their experience during and when recalling an event, and the accessibility of events when asked to recall an AM. Regarding the experience of positive and negative events, Klaiber et al. (2022) found that not only higher individual levels of extraversion but also openness was associated with an increased likelihood for an individual to report having experienced positive events. In accordance, in the present data, openness was associated with a higher positive intensity of individuals' positive AMs. Furthermore, similar to extraversion, also openness, agreeableness, and conscientiousness have been found to be associated with adaptive emotion regulation strategies such as cognitive reappraisal (Barańczuk 2019), which in turn has been found to be associated with higher positive affect intensity of AMs (Wisco and Nolen‐Hoeksema 2010). Fittingly, in the present data, both agreeableness and conscientiousness were associated with a higher positive affect intensity of individuals' negative AMs. Finally, previous research has shown that not only extraversion, but also openness, agreeableness, and conscientiousness are associated with different aspects of dispositional positive affect (Shiota et al. 2006), and a number of previous studies have demonstrated that momentary affect is associated with the accessibility of emotionally congruent memories (e.g., Faul and LaBar 2022). These findings and theoretical considerations lead to the assumption that, in addition to the effects of neuroticism and extraversion, openness, agreeableness, and conscientiousness should all be associated with more positive and/or less negative affect intensity of individuals' positive and/or negative AMs—a pattern that was confirmed in the present data.

In summary, previous empirical evidence suggests that neuroticism and to some extent extraversion, but not openness, agreeableness and conscientiousness are related to the affect intensity of individuals' AMs. However, this evidence comes from a small number of studies, with rather small sample sizes or specific restrictions on the selection of AMs. The present findings are consistent with these studies with respect to neuroticism and extraversion, but in terms of openness, agreeableness and conscientiousness, the present results show a pattern that is more consistent with the predictions derived from the theoretical perspectives on the association outlined in the introduction to this article. Future studies might replicate these findings in order to strengthen our understanding of the relationship between the Big Five and the affect intensity of individuals' positive and negative AMs.

## 10 Limitations and Future Directions

A limitation of the present study is that the BFI‐K does not contain facets. It may be revealing to investigate whether specific facets of personality domains are particularly relevant in relation to the positive and negative affect intensity of individuals' AMs. Future studies should examine the association of the Big Five and their facets with the positive and negative affect intensity of individuals' AMs using more detailed scales to measure the Big Five, such as the NEO‐PI‐R (Costa and McCrae 1992).

Another limitation of the present study is that only three positive and three negative AMs were collected per participant. In particular, because of their salience, it is very likely that most of the memories recalled were highly intense and important. Indeed, as can be seen in Table 2, the recalled events were on average rated as highly intense, but this effect slowly diminished from the first to the third memory, suggesting that sampling a larger number of AMs may have sampled less emotionally intense memories as well. Future studies may replicate the present study design but collect more AMs per participant to investigate whether the effects still hold for less important and less salient events. Moreover, future studies could also examine the effects of emotional order in relation to personality (e.g., Nusser et al. 2024). For instance, neuroticism may be related not only to less positive affect intensity of individuals' positive AMs, but perhaps also to a greater ‘decline’ in the positive intensity across the positive events reported by an individual.

Moreover, although the theoretical considerations of the present article were largely based on previous findings of Big Five differences in experiencing and perceiving specific events, as well as emotion‐regulation and dispositional affect, the present study did not directly examine any of these variables in relation to the association between the Big Five and the affect intensity of individuals' AMs. In order to better understand why and through what mechanisms personality is associated with individuals' autobiographical memory, future studies could, for instance, include mood or dispositions to certain emotion regulation strategies in their analyses as mediators of the association. In addition, consistent with the idea that emotion regulation to some extent alters the affect intensity associated with an AM, future studies could examine how the Big Five relate to perceived affect changes in the positive and negative affect intensity of individuals' AMs (e.g., Hoehne 2023; Hoehne and Zimprich 2024, 2025b).

In this regard, it should be pointed out that the theoretical considerations of the relationship between personality traits and AM affect intensity presented here do not claim to be exhaustive. There may be many other mechanisms by which personality might be related to the affect intensity of AMs. One possibility is that dispositions to different personality traits may be differentially related to memory specificity, which in turn may influence the reported affect intensity of AMs. In particular, high levels of neuroticism may be associated with less specific AMs, given that psychopathology, such as in the form of depression, has often been linked to overgeneral memory (e.g., Wilson and Gregory 2018). Future studies may seek to test different possible explanations for the association between personality traits and the affect intensity of individuals' AMs.

Finally, the present sample consisted mainly of well‐educated individuals whose first language was German, which should be taken into account when interpreting the present results. Future studies may attempt to replicate these findings in different samples and cultures.

## 11 Conclusion

In conclusion, the present study provides further empirical evidence for the association between neuroticism and the positive and negative affect intensity of individuals' positive and negative AMs, while extending the evidence for extraversion. In addition, the present study demonstrates that openness, agreeableness and conscientiousness are also related to the affect intensity of individuals' AMs, thereby further extending previous research. Finally, in addition to reviewing previous empirical evidence, the present manuscript describes a theoretical basis for why and in which direction different personality traits may be related to the affect intensity of individuals' AMs, which predictions, although not directly tested, were supported by the present findings.

## Author Contributions

**Sophie Hoehne:** conceptualization, writing – original draft, writing – review and editing, formal analysis, methodology.

## Ethics Statement

This study did not meet the criteria proposed by the ethics committee of Ulm University for a full application for ethical approval.

## Conflicts of Interest

The author declares no conflicts of interest.

## Supporting information


**Table S1:** Bivariate correlations between BFI‐K items (*N* = 1275).

## Data Availability

Data and analysis code are available in an Open Science Framework repository at https://doi.org/10.17605/OSF.IO/72W5M.

## Endnotes

^1^
A comparison of those 419 persons excluded due to missing AM data with these 1275 participants showed that the two groups did not differ significantly with respect to the Big Five. In line with this, effect sizes were minimal (extraversion: *d* = −0.08, agreeableness: *d* = −0.07, conscientiousness: *d* = 0.01, neuroticism: *d* = 0.04, openness to experience: *d* = −0.10; with negative values denoting that those with missing AM data had lower values).

^2^
As multilevel models are rather complex, conducting an a priori power analysis for multilevel models is complicated and unambiguous conclusions are difficult to make in many cases (Kreft and Leeuw 1998). The sample size of the present study was guided by general suggestions for the use of multilevel models (e.g., Maas and Hox 2005) and multiple regression (Maxwell 2004). Although no a priori power analysis was conducted, the sample size of 1275 participants is sufficient to assume adequate power.

^3^
The study was conducted in Germany. Participants therefore indicated whether they had completed the German Abitur (comprising 12 to 13 years of schooling). I refer to this as “university entrance diploma” in the present manuscript.

^4^
Other questionnaires that were collected but not included in the present study are the Brief Resilience Scale (Smith et al. 2008), Herth Hope Index (Herth 1992), Life Orientation Test (Scheier and Carver 1985), Rosenberg Self‐Esteem Scale (Rosenberg 1965), the Temporal‐Focus Scale (Shipp et al. 2009), the SF‐12 (Ware et al. 1996), the Short Form of the Revised Almost Perfect Scale (Rice et al. 2014), the Future Time Perspective Scale (Carstensen and Lang 1996) and the Temporal Satisfaction With Life Scale (Pavot et al. 1998).

^5^
Usually, a cut‐off value of 0.70 is chosen for Cronbach's alpha. However, given the small number of items per factor in the BFI‐K, previous studies have found that the internal consistency for some factors is sometimes slightly lower (Kovaleva et al. 2014; Rammstedt and John 2005), so the cut‐off value was set at 0.60.

^6^
We added the instruction that the events did not have to be extraordinary in order to ensure that participants did not think that any of their spontaneous memories were not important enough to be reported in the study. Moreover, it was added to the instruction that the reported events should be more than 1 year old. This was done because it takes time for recent memories to be integrated into the long‐term memory system, that is to become autobiographical memories (Conway 2005).

^7^
Other questions, not used for the present analysis included age at the time of the event, positive and negative affect intensity from the perspective of the event at the time it happened, and whether the event had changed in affect intensity or valence from then (occurrence) to now (test).

^8^
The affect intensity items had a Likert‐type scale response format. In addition, because of the memory cueing method, that is free recall of a given number of positive and negative events, ceiling effects in the distribution of responses are likely. To account for both cases, all analyses were conducted treating the affect intensity ratings as ordered‐categorical. The results did not differ from the models in which the affect intensity ratings were treated as linear. Therefore, in order to reduce the complexity of the present analysis, I decided to report the results for linear affect intensity ratings.

^9^
Some recent studies on the Big Five have used Exploratory Structural Equation Modeling (ESEM) instead of EFA (e.g., Marsh et al. 2010). Indeed, ESEM has many advantages over classical EFA, such as providing goodness of fit indices (cf., Asparouhov and Muthén 2009). However, the joint application of multilevel models with ESEM is rather limited (e.g., Swami et al. 2023; van Zyl and ten Klooster 2022), so we retained classical EFA for the present analysis.

^10^
Participants who identified as gender diverse or did not wish to disclose their gender were given a value of 0 in the centered binary gender variable, so that they would add to the effects of all predictor variables except gender.

^11^
To test for potential suppression effects, I also regressed correlated Big Five traits on the affect intensity of the AMs, and found similar negative effects of conscientiousness as Blagov et al. (2022). Further analyses revealed that this effect was indeed due to negative suppression.
