# Supplementary material for: Big Five Personality Factors Differentially Related to Positive and Negative Affect Intensity of Autobiographical Memories
Source: Scand J Psychol. 2025 Oct 30;67(2):413–25. doi: 10.1111/sjop.70039 (PMC12983994; doi:10.1111/sjop.70039)
Supplement: Supplementary file 1 — Table S1: Bivariate correlations between BFI‐K items (N = 1275). [file SJOP-67-413-s001.docx]

**Online Supplemental Material for “Big Five Personality Factors Differentially Related to Positive and Negative Affect Intensity of Autobiographical Memories”**

### **Exact Wording of Instructions for Retrieving Autobiographical Memories**

*General instruction:* “Autobiographical memory contains memories of events that you have experienced yourself. Your task will be to describe three memories of experiences from your past. These do not have to be anything special or spectacular, but simply experiences that you remember. It is important that these are specific events that happened a little further back in time (at least one year ago).”

*Specific instruction for positive AMs:* “The three memories we are asking you to recall should all relate to events that you experienced as rather positive at the time they happened. Positive events can also have negative aspects. Therefore, do not be surprised if you are also asked about negative feelings in connection with positive experiences.”

*Specific instruction for negative AMs:* “The three memories we are asking you to recall should all relate to events that you experienced as rather negative at the time they happened. Negative events can also have positive aspects. Therefore, do not be surprised if you are also asked about positive feelings in connection with negative experiences.”

**Table S1**

*Bivariate Correlations between BFI-K Items (N = 1275)*

| Measure | 1 | 2 | 3 | 4 | 5 | 6 | 7 | 8 | 9 | 10 | 11 |
| --- | --- | --- | --- | --- | --- | --- | --- | --- | --- | --- | --- |
| *Neuroticism* | | | | | | | | | | | |
| 1. Item 4 | 1.00 |  |  |  |  |  |  |  |  |  |  |
| 2. Item 9 ^r^ | 0.52* | 1.00 |  |  |  |  |  |  |  |  |  |
| 3. Item 14 | 0.62* | 0.49* | 1.00 |  |  |  |  |  |  |  |  |
| 4. Item 19 | 0.60* | 0.53* | 0.57* | 1.00 |  |  |  |  |  |  |  |
| *Extraversion* | | | | | | | | | | | |
| 5. Item 1 ^r^ | -0.28* | -0.09* | -0.26* | -0.36* | 1.00 |  |  |  |  |  |  |
| 6. Item 6 | -0.21* | -0.15* | -0.13* | -0.25* | 0.42* | 1.00 |  |  |  |  |  |
| 7. Item 11 ^r^ | -0.24* | -0.06* | -0.18* | -0.31* | 0.78* | 0.43* | 1.00 |  |  |  |  |
| 8. Item 16 | -0.28* | -0.20* | -0.23* | -0.32* | 0.67* | 0.51* | 0.61* | 1.00 |  |  |  |
| *Openness* | | | | | | | | | | | |
| 9. Item 5 | -0.17* | -0.14* | -0.09* | -0.13* | 0.10* | 0.30* | 0.09* | 0.19* | 1.00 |  |  |
| 10. Item 10 | 0.15* | 0.10* | 0.26* | 0.12* | -0.08* | 0.12* | -0.06* | -0.03 | 0.26* | 1.00 |  |
| 11. Item 15 | -0.02 | -0.02 | 0.07* | -0.04 | 0.05 | 0.28* | 0.07* | 0.09* | 0.40* | 0.38* | 1.00 |
| 12. Item 20 | 0.04 | 0.01 | 0.06* | 0.01 | 0.05 | 0.18* | 0.07* | 0.09* | 0.34* | 0.24* | 0.38* |
| 13. Item 21 ^r^ | 0.02 | 0.03 | 0.04 | -0.01 | 0.04 | 0.15* | 0.06* | 0.07* | 0.35* | 0.26* | 0.44* |
| *Agreeableness* | | | | | | | | | | | |
| 14. Item 2 ^r^ | -0.16* | -0.13* | -0.14* | -0.10* | 0.01 | 0.06* | -0.01 | 0.05 | 0.05 | 0.03 | 0.04 |
| 15. Item 7 | -0.10* | -0.08* | -0.06* | -0.02 | 0.12* | 0.23* | 0.10* | 0.23* | 0.11* | 0.09* | 0.12* |
| 16. Item 12 ^r^ | -0.14* | -0.04 | -0.15* | -0.06* | 0.20* | 0.10* | 0.20* | 0.18* | -0.01 | -0.04 | -0.01 |
| 17. Item 17 ^r^ | -0.16* | -0.07* | -0.12* | -0.04 | 0.12* | 0.06* | 0.09* | 0.13* | 0.02 | -0.01 | -0.01 |
| *Conscientiousness* | | | | | | | | | | | |
| 18. Item 3 | -0.11* | -0.10* | 0.03 | -0.10* | 0.02 | 0.14* | 0.00 | 0.09* | 0.14* | 0.15* | 0.04 |
| 19. Item 8 ^r^ | -0.26* | -0.06* | -0.19* | -0.25* | 0.20* | 0.19* | 0.17* | 0.15* | 0.15* | 0.00 | 0.01 |
| 20. Item 13 | -0.17* | -0.12* | -0.08* | -0.17* | 0.09* | 0.20* | 0.08* | 0.15* | 0.13* | 0.04 | 0.05 |
| 21. Item 18 | -0.20* | -0.17* | -0.14* | -0.19* | 0.13* | 0.25* | 0.13* | 0.22* | 0.20* | 0.10* | 0.10* |

*Note*. *p < .05; ^r^ indicates that the original item was reversed.

**Table S1 (continued)**

*Bivariate Correlations between BFI-K Items (N = 1275)*

| Measure | 12 | 13 | 14 | 15 | 16 | 17 | 18 | 19 | 20 | 21 |
| --- | --- | --- | --- | --- | --- | --- | --- | --- | --- | --- |
| *Neuroticism* | | | | | | | | | | |
| 1. Item 4 |  |  |  |  |  |  |  |  |  |  |
| 2. Item 9 ^r^ |  |  |  |  |  |  |  |  |  |  |
| 3. Item 14 |  |  |  |  |  |  |  |  |  |  |
| 4. Item 19 |  |  |  |  |  |  |  |  |  |  |
| *Extraversion* | | | | | | | | | | |
| 5. Item 1 ^r^ |  |  |  |  |  |  |  |  |  |  |
| 6. Item 6 |  |  |  |  |  |  |  |  |  |  |
| 7. Item 11 ^r^ |  |  |  |  |  |  |  |  |  |  |
| 8. Item 16 |  |  |  |  |  |  |  |  |  |  |
| *Openness* | | | | | | | | | | |
| 9. Item 5 |  |  |  |  |  |  |  |  |  |  |
| 10. Item 10 |  |  |  |  |  |  |  |  |  |  |
| 11. Item 15 |  |  |  |  |  |  |  |  |  |  |
| 12. Item 20 | 1.00 |  |  |  |  |  |  |  |  |  |
| 13. Item 21 ^r^ | 0.65* | 1.00 |  |  |  |  |  |  |  |  |
| *Agreeableness* | | | | | | | | | | |
| 14. Item 2 ^r^ | 0.05 | 0.13* | 1.00 |  |  |  |  |  |  |  |
| 15. Item 7 | 0.12* | 0.08* | 0.23* | 1.00 |  |  |  |  |  |  |
| 16. Item 12 ^r^ | 0.02 | 0.08* | 0.32* | 0.26* | 1.00 |  |  |  |  |  |
| 17. Item 17 ^r^ | 0.05 | 0.10* | 0.45* | 0.23* | 0.64* | 1.00 |  |  |  |  |
| *Conscientiousness* | | | | | | | | | | |
| 18. Item 3 | 0.01 | -0.02 | 0.04 | 0.08* | 0.05 | 0.05 | 1.00 |  |  |  |
| 19. Item 8 ^r^ | 0.03 | 0.06* | 0.19* | 0.02 | 0.20* | 0.16* | 0.35* | 1.00 |  |  |
| 20. Item 13 | 0.01 | -0.02 | 0.02 | 0.07* | 0.03 | 0.02 | 0.48* | 0.41* | 1.00 |  |
| 21. Item 18 | 0.06* | 0.04 | -0.03 | 0.09* | 0.05 | -0.01 | 0.36* | 0.38* | 0.40* | 1.00 |

*Note*. *p < .05; ^r^ indicates that the original item was reversed.
